# Predicting mortality in patients with nonvariceal upper gastrointestinal bleeding using machine-learning

**DOI:** 10.3389/fmed.2023.1134835

**Published:** 2023-02-17

**Authors:** Bogdan Silviu Ungureanu, Dan Ionut Gheonea, Dan Nicolae Florescu, Sevastita Iordache, Sergiu Marian Cazacu, Vlad Florin Iovanescu, Ion Rogoveanu, Adina Turcu-Stiolica

**Affiliations:** ^1^Department of Gastroenterology, University of Medicine and Pharmacy of Craiova, Craiova, Romania; ^2^Department of Pharmacoeconomics, University of Medicine and Pharmacy of Craiova, Craiova, Romania

**Keywords:** UGIB, Rockall score, Beylor Bleeding score, machine learning, Glasgow Blatchford score

## Abstract

**Background:**

Non-endoscopic risk scores, Glasgow Blatchford (GBS) and admission Rockall (Rock), are limited by poor specificity. The aim of this study was to develop an Artificial Neural Network (ANN) for the non-endoscopic triage of nonvariceal upper gastrointestinal bleeding (NVUGIB), with mortality as a primary outcome.

**Methods:**

Four machine learning algorithms, namely, Linear Discriminant Analysis (LDA), Quadratic Discriminant Analysis (QDA), logistic regression (LR), K-Nearest Neighbor (K-NN), were performed with GBS, Rock, Beylor Bleeding score (BBS), AIM65, and T-score.

**Results:**

A total of 1,096 NVUGIB hospitalized in the Gastroenterology Department of the County Clinical Emergency Hospital of Craiova, Romania, randomly divided into training and testing groups, were included retrospectively in our study. The machine learning models were more accurate at identifying patients who met the endpoint of mortality than any of the existing risk scores. AIM65 was the most important score in the detection of whether a NVUGIB would die or not, whereas BBS had no influence on this. Also, the greater AIM65 and GBS, and the lower Rock and T-score, the higher mortality will be.

**Conclusion:**

The best accuracy was obtained by the hyperparameter-tuned K-NN classifier (98%), giving the highest precision and recall on the training and testing datasets among all developed models, showing that machine learning can accurately predict mortality in patients with NVUGIB.

## Introduction

Upper gastrointestinal bleeding (UGIB) still represents a common cause of gastroenterological admission and usually requires risk stratification for the level of care determination as well as rapid decision management (1). In order to differentiate the high-risk groups of patients in the emergency department, multiple guidelines developed pre-endoscopic risk assessment scores which combine both clinical features and biological parameters (2). Both the American Society of Gastroenterology (ASGE) and the European Society of Gastrointestinal Endoscopy (ESGE) recommend Glasgow-Blatchford (GBS), Rockall admission score (Rock), and AIM65 as possible tools to assess UGIB patients on their first presentation (3, 4). However, some studies suggested that the most accurate score for patient risk differentiation is GBS with multiple outcomes such as necessary transfusions, endoscopic reintervention, and death (5, 6).

Postponing endoscopy is also recommended whenever low-risk patients are identified, however, delaying endoscopy could also lead to dramatic consequences if patient selections are not done well. Several studies have proposed a hierarchy of patients who present with UGIB by defining patients that might be delayed till endoscopy (7, 8). While many centers, still need to reschedule endoscopy until the next morning or over the weekend, new methods should be proposed for a better discerning of patient’s evolution.

Artificial intelligence in gastroenterology is on continuous path-breaking development, especially on imaging recognition patterns with already proposed techniques for daily practice (9, 10). The term AI covers machine learning (ML) and specific techniques such as deep learning (DL) by using data sets for pattern recognition by combining several variables which will further allow transposing new data that uses the same variables. Available clinical models for UGIB allow patients’ features and predictors to suggest the prognostic. By involving an artificial neural network (ANN), the data trained to determine the desired outcome may be used to predict the output on input data of newly identified cases that may be encountered. Thus, by doing a repetitive learning technique, the ANN will be able to foretell the outcomes of the patient’s prognosis.

The development of new models of patient triage and follow-up should be promoted to reduce medical exposure, thus managing possible complications. Moreover, by using ANN the results might be even more effective since the human factor is bypassed. The patient’s prognosis presented with non-variceal UGIB (NVUGIB) should be assessed as early as possible in order to determine the proper timing of endoscopy. The aim of our study was to provide a new ANN that sums up all available pre-endoscopic risk scores for patients with UGIB for predicting mortality, thus promoting patients for new endoscopic procedures or even surgery.

## Materials and methods

### Patients

The Ethics Committee of the University of Medicine and Pharmacy of Craiova, Romania approved this retrospective study and informed consent from all patients were acquired in the County Hospital before patient enrolment in the study (11977/24.03.2020). We selected 1,096 patients who were admitted for UGIB from March 2018 to December 2021within the Gastroenterology Department of the Emergency County Hospital of Craiova, Romania. The selection was based on the criteria: (1) patients with NVUGIB, (2) age ≥ 18 years old, (3) existing information as mortality, GBS, Rock, Beylor Bleeding score (BBS), AIM65, and T-score. Furthermore, the following exclusion criteria were considered: (1) patients with variceal UGIB, (2) patients with any type of cancer, (3) patients with important missing data (for example, data for calculating the scores).

### Machine learning analysis framework

We adopted multiple machine-learning (ML) models, including Linear Discriminant Analysis (LDA), Quadratic Discriminant Analysis (QDA), logistic regression (LR), K-Nearest Neighbor (KNN). We tried GridSearch, RandomSearch as model tuning techniques to see if that improves the model’s final performance. Confusion matrix was used to check model performance with or without standardization and we recorded accuracy, precision, recall, and f1-score. These classifiers were compared in terms of predicting the likelihood of mortality. The data was split into 70% train and 30% test sets, using the stratified sampling technique to ensure that relative class frequencies are approximately preserved in each train and validation fold. We used descriptive statistics to summarize the patients’ characteristics: counts (percentages) for categorical variables and mean ± standard deviation (SD) for continuous variables.

The models predicted whether a NVUGIB patient would experience mortality by learning a number of five clinical scoring systems: GBS, Rock, BBS, AIM65, and T-score. Covariance matrix was introduced in the equation to consider the variation among the independent variables (GBS, Rock, BBS, AIM65, T-score). The ROC curve (receiver operating characteristic curve) and the area under this curve (AUC) for every single scoring system were used to quantify the visual profile of the ability of a model that includes only one score.

The confusion matrix shows clockwise from top left: True Negative (TN, model predicts that a NVUGIB patient would live and the patient does not die), False Positive (FP, model predicts that a NVUGIB patient would die but the patient actually does not die), True Positive (TP, model predicts that a NVUGIB patient would die and the patient dies) and False Negative (FN, model predicts that a NVUGIB patient would live but the patient actually dies). The recall [the fraction of total actually positive cases that are predicted correct = TP/(TP+FN)] will predict the need for intervention without high westing of hospital resources. It is preferred to use recall because the healthcare system cannot afford to make false-negative errors. The greater the Recall, the higher the chances of minimizing FN. Precision is the fraction of total positive predictions that are actually correct [TP/(TP+FP)]. F1-score is used when both precision and recall seem to be important.

Linear Discriminant Analysis draws one hyperplane and projects the data onto this hyperplane in such a way as to maximize the separation of the patients who died, according to two criteria: maximizing the distance between the means of the two classes and minimizing the variation between each category (11).

Quadratic Discriminant Analysis is a probabilistic parametric classification technique that represents an evolution of LDA for nonlinear class separations. QDA, like LDA, is based on the hypothesis that the probability density distributions are multivariate normal but, in this case, the dispersion is not the same for all of the categories (12).

Logistic regression is a supervised learning algorithm where we used the sigmoid function to calculate the probability of dying given the five scores, using also Lasso regularization (13).

K-Nearest Neighbor is a non-parametric algorithm, it does not make any assumption on underlying data. Because all the variables are continuous, we can apply LDA, assuming normality assumption for P(X|Y = 1) and P(X|Y = 0), and homoscedasticity (the covariance matrices are equal among the 2 classes) and QDA if the class variance are not the same (14).

### Statistical analysis

The models were implemented using an open-source program language (Python 3.7.1), using its packages (numpy, scikit-learn, matplotlib). Continuous numerical variables were expressed as means (± standard deviation) and median (interquartile range, 25% quantile–75% quantile) and categorical variables were expressed as percentages. We used the Mann–Whitney U test for continuous variables. The *p*-value less than 0.05 was significant.

## Results

### Patients characteristics

This study implied 1,096 patients with NVUGIB (738 men, 67.3%; mean age ± SD, 63.9 ± 14.6). Socio-demographic and clinical features of patients are shown in Table 1. A percentage of 11% of these patients had cirrhosis and 7.5% mortality was registered.

**TABLE 1:** Socio-demographic and clinical characteristics of the study subjects.

Characteristics	Frequency in the population study (*N* = 1096)*
Age (years), mean ± SD, range	63.9 ± 14.6, 17–92
Gender, male	738 (67.34%)
Urban residence	530 (48.36%)
Hospital days	8 ± 7.2
Mortality	82 (7.48%)
Rebleeding	32 (2.92%)
Surgery	11 (1%)
Hematemesis	472 (43.07%)
Platelets (no/mcL)	211,096.1 ± 97,621.5
Creatinine (mg/dL)	1.2 ± 1.3
Cirrhosis, yes	121 (11.04%)
**Comorbidities**	
Cardiovascular diseases	65 (5.93%)
Chronic kidney diseases	34 (3.1%)

*Continuous variables are expressed in mean ± SD and discrete variables are expressed in frequency and percentages.

### Performance of models and classifiers

The five scores for the class groups of mortality are summarized in Table 2 and no significant differences were observed only for BBS (*p*-value = 0.099). The values for GBS, Rock, and AIM65 were significantly higher, and T-score was significantly lower for patients that died.

**TABLE 2:** Statistical characteristics of the two groups divided by the mortality in the study population.

Characteristics	Total (*n* = 1096)	Mortality		*p*-value
		No (*n* = 1014)	Yes (*n* = 82)	
Glasgow Blatchford	9.9 ± 3.6 10 (8–12)	9.76 ± 3.56 10 (8–12)	12.26 ± 3.2 12 (10–14.25)	<0.001
Rockall score	3.7 ± 1.9 4 (2–5)	3.64 ± 1.89 4 (2–5)	4.34 ± 1.74 4 (4–5)	0.001
Beylor Bleeding score	7.6 ± 4.1 8 (4–11)	7.5 ± 4.14 8 (3.75–11)	8.32 ± 3.93 8.5 (6–11)	0.099
AIM65	1.1 ± 0.9 1 (1–1)	1.02 ± 0.83 1 (0-1)	1.8 ± 1.05 1 (1–2.25)	<0.001
T-score	9.3 ± 2.0 9 (8–10)	9.34 ± 2.01 9 (8–11)	8.85 ± 1.91 9 (7–9.25)	0.024

Data are presented as mean ± SD and median (interquartile range).

**FIGURE 1:**
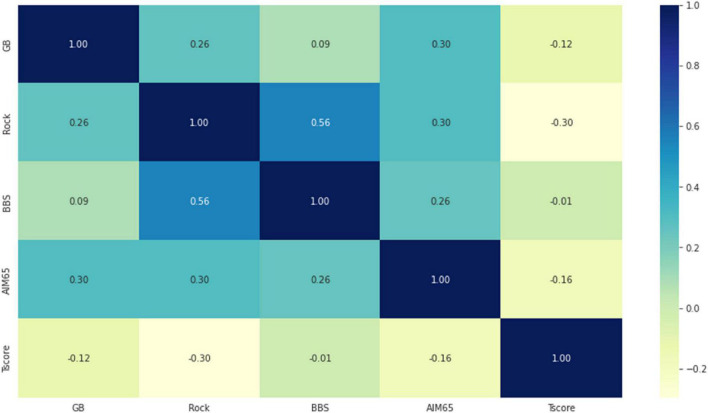
The correlation heatmap between measured scores (colors range from dark blue for strong positive correlations, to bright yellow, for strong negative correlations).

Statistically significant correlations were found between the five scores, even if they are low or very low, as in Figure 1. Positive correlation coefficients were observed, except by making the T-score where they were negative correlated (rho for T-score and GBS = −0.12, *p*-value < 0.001; rho for T-score and Rock = −0.30, *p*-value < 0.001; rho for T-score and AIM65 = −0.16, *p*-value < 0.001). the strongest correlation was observed between Rock and BBS (rho = 0.56, *p*-value < 0.001).

**FIGURE 2:**
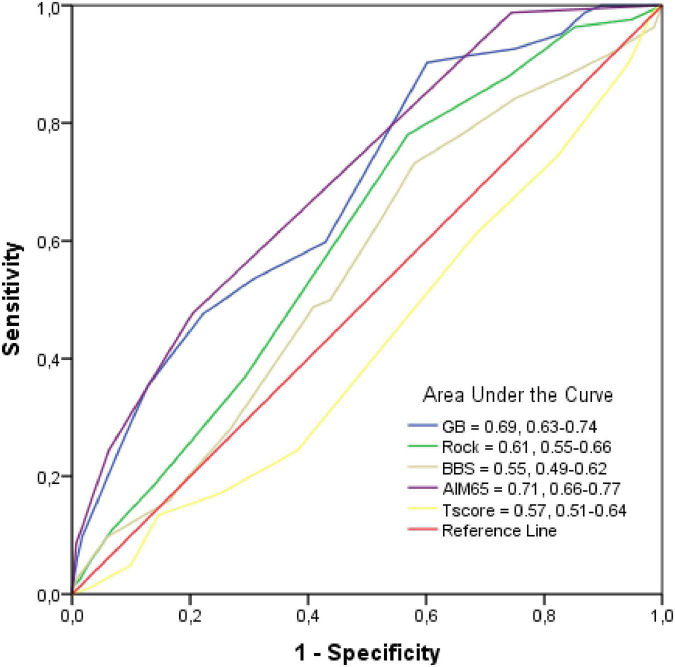
Receiver operating characteristic (ROC) Curve and Area under the curve (AUC). Mean AUC and its 95% confidence interval of the scores are shown in the legends of the subplots.

The AUC for GBS, Rock, BBS, AIM65, and T-score was low, the highest value was observed for AIM65 (AUC = 0.71, 95% CI: 0.66–0.77), as in Figure 2.

Classification accuracy of each machine-learning model (LDA, QDA, LR, and K-NN) was evaluated and summarized in Table 3.

**TABLE 3:** Comparison of the confusion matrix and evaluation measures among prediction models.

	Precision	Recall	F1-score	Support
**LDA**				
Survival	0.95	0.95	0.95	709
Death	0.32	0.31	0.32	54
Accuracy			0.90	763
Macro avg	0.63	0.63	0.63	763
Weighted avg	0.90	0.90	0.90	763
**QDA**				
Survival	0.94	0.97	0.95	709
Death	0.32	0.19	0.24	54
Accuracy			0.91	763
Macro avg	0.63	0.58	0.60	763
Weighted avg	0.90	0.91	0.90	763
**LG**				
Survival	0.95	0.95	0.95	709
Death	0.32	0.28	0.30	54
Accuracy			0.91	763
Macro avg	0.63	0.62	0.62	763
Weighted avg	0.90	0.91	0.90	763
**K-NN**				
Survival	0.98	1.00	0.99	709
Death	1.00	0.78	0.88	54
Accuracy			0.98	763
Macro avg	0.99	0.89	0.93	763
Weighted avg	0.98	0.98	0.98	763

The reported average includes the macro average which averages the unweighted mean per label, and the weighted average which averages the support-weighted mean per label.

**FIGURE 3:**
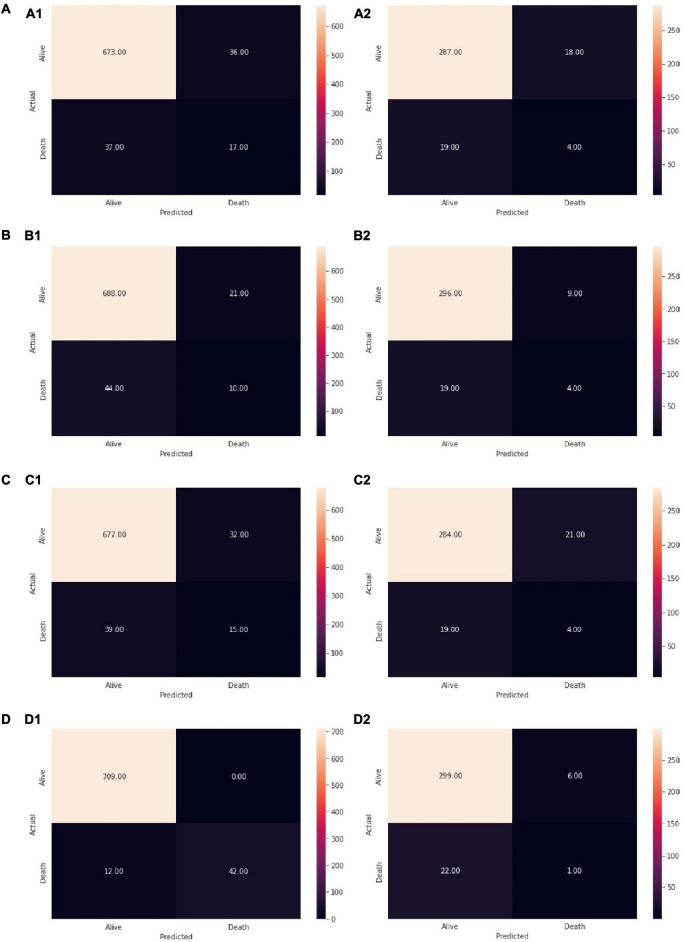
Checking model performance of **(A1)** LDA on training data, **(A2)** LDA on test data, **(B1)** QDA on training data, **(B2)** QDA on test data, **(C1)**. LR on training data, **(C2)** LR on test data, **(D1)** K-NN on training data, **(D2)** K-NN on test data. Reading the confusion matrix (clockwise from top left): True Negative (Actual = Alive, Predicted = Alive): Model predicts that the patient would live and the patients’ lives, False Positive (Actual = Alive, Predicted = Death): Model predicts that the patient would die and the patients actually lives, True Positive (Actual = Death, Predicted = Death): Model predicts that the patient would die and the patients dies, False Negative (Actual = Death, Predicted = Alive): Model predicts that the patient would live and the patients actually dies.

The LDA model is performing well in terms of accuracy on the training data, as in Figure 3A1. The recall for death is quite low (0.31), which implies that this model will not perform well in differentiating the patients who have a high chance of survival, and hence this model would not help reduce the mortality rate. The model is giving a decent average recall when we balanced the precision and the recall for a threshold of about 0.25. A recall of 0.63 suggests that there is a 37% chance that the model will predict that a person is going to die even though he/she would not, and the health system would waste their time and money on these patients who are not at risk of mortality. We have built the LDA model. Furthermore, checking the coefficients, we found which variables are leading to mortality and which can help to reduce the mortality. The scores which positively affect the mortality are AIM65 (coefficient = 0.93) and GBS (coefficient = 0.57) and the ones that negatively affect it are T-score (coefficient = –0.31) and Rock (coefficient = –0.25). Based on LDA model, AIM65 is the most important feature in detecting whether a NVUGIB patient would die or not and BBS has almost no effect in predicting this (coefficient = 0.04). We checked the performance on the test data in Figure 3A2. The model was giving a similar performance on the test and train data, meaning the model has generalized well. The average recall, the precision and the accuracy are good, but we evaluated if we could get a better performance using other algorithms.

The QDA model did not obtained different outcomes from the LDA model (even worse recall), as in Figure 3B.

The LR model was giving a similar performance on the test and the train datasets (Figure 3C). The recall of the test data has increased while at the same time, the precision has decreased slightly, which was to be expected while adjusting the threshold at 0.18. The accuracy was of 0.91 on the train and of 0.90 on the test datasets. Checking the coefficients of the model, we observed the same variables that are leading to mortality rate: AIM65 (coefficient = 0.60) and GBS (coefficient = 0.57) and which can help to deduce the mortality rate: T-score (coefficient = −0.17) and Rock (coefficient = −0.21). The coefficients that positively and negatively affect the mortality rate were similar for LR and LDA. This means they capture the same pattern and give the same conclusions from the dataset.

**FIGURE 4:**
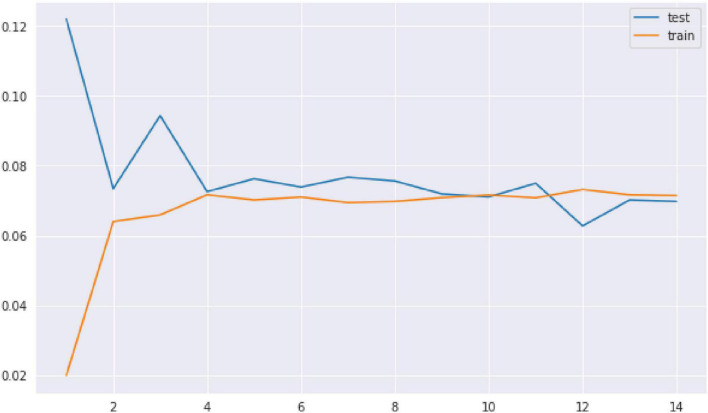
Test and train error for the K-NN model. As the number of neighbors increases, the test error and the train error are the same.

Performing the KNN model from the Figure 3D, we selected the best value of k for which the error rate is the least in the validation data and k = 14 gave us the generalized model with very similar train and test errors, as in Figure 4.

We used GridSearchCV for hyperparameter tuning and we used them to find a better recall of the model. The recall and the precision have significantly increased by tuning the K-NN classifier. This is a high-performing model that a physician can use to control the mortality rate. There is a 98% chance that the model will detect NVUGIB patients who are likely to die, and the physician can take the appropriate action.

## Discussion

Patient stratification in UGIB has been considered for prognosis assessment by differentiating high-risk patients (15). So far, available prediction scores use only some variables, both clinical or biological, and are based on conventional statistical analysis. While some of them are used for rebleeding or death prediction, a high precision rate has not been achieved. Ensuring a risk stratification at patient admission might be helpful in choosing the proper time for endoscopy, especially in small regional hospitals which do not provide a full-time endoscopy service. Moreover, probably a turning point in medicine in the last years, the COVID-19 pandemic almost changed patients’ presentation in the emergency room, as well as patients’ admission (16). A general decrease in patients’ admission has been observed in the first months of the pandemic for all types of disease, and also for UGIB patients. While the first consideration was that endoscopy was a high-risk procedure and should be performed only if patients required it, due to the lack of medical materials as well as the fear of contamination or hospital circuit reorganization, many patients still required rapid endoscopic assessment due to UGIB (17). Providing a tool to delay endoscopy or to predict the death secondary to UGIB might organize better the endoscopist decision-making process in choosing the right time for endoscopy.

European Society of Gastrointestinal Endoscopy updated guidelines on NVUGIB recommend the use of GBS as the main risk stratification after patient admission (18). As stated, patients with a GBS ≤ 1 may be successfully managed as outpatients and may be discharged, however, patients require to be notified of the possibility of rebleeding, thus they should maintain contact with the discharging hospital. When discussing high-risk patients, there is a low probability of discharging and GBS score has shown a specificity of 12% for transfusions, hemostatic interventions as well as death (19). Also, when NVUGIB is associated with liver cirrhosis, mortality might increase due to the underlying disease complications such as hepatic encephalopathy and spontaneous bacterial peritonitis (20).

Our study provides a non-endoscopic ML model as an alternative tool to predict mortality in patients with NVUGIB at their admission to the emergency department. We obtained a high accuracy for death prediction and surpassed the available scores used for the initial assessment. There are some other studies that used AAN to predict mortality in UGIB, showing also better results than the current clinical scores (21–24). Available studies suggest that risk assessment tools have an AUC of 0.77 for mortality as mentioned in two multicenter studies (25, 26). However, our study points out that ANN might be more efficient in highlighting patients’ prognoses related to mortality, with an AUC of 0.99. Moreover, the results are even more optimistic than the available ANN used so far for UGIB assessment by ANN or ML models.

A systematic review showed that ML models were more effective in predicting rebleeding, intervention, and mortality, with an AUC ranging from 0.80 to 0.90 (27). The ANN we propose focuses on five non-endoscopic scores used as an initial assessment to stratify the risk of UGIB. We combined GBS, AIMS65, Rock, T-score as well as BBS in a ML model, thus trying to better identify patients with a dismal prognosis. Our study end-point was mortality as we focused on exploring the potential of all five scores combined within a newly developed ML. Noteworthy is that taken separately all risk scores were definitely less accurate than our prediction model. Thus, our model might enable new opportunities for non-invasive tools to predict the NVUGIB mortality rate.

Risk assessment represents a cornerstone for the healthcare system, as it may provide high-quality care for patients and may also help save resources and direct them to more precise interventions. Even though there is a long distance to implementing this type of model in clinical practice, the potential of ML for UGIB assessment should not be downplayed (28, 29). We do acknowledge that it may be challenging to transfer an ML to a clinical setting, however, AI depicting background may attempt to integrate into clinical care and provide more reliable measures for UGIB assessment.

Nonetheless, our study has certain limitations. Firstly, this is a single-center experience study, thus we validated our AAN only on patients admitted to our Clinic. Secondly, we had a small sample size, but without missing data, and the Precision and Recall obtained in the validation dataset were not low. Finally, we prepared our dataset from the retrospective database, but the outcomes could not have changed over time due to the update of treatment guidelines in the last years. Testing the algorithm in a multicenter setting will surely help validate and improve our objective. On the other hand, we focused only on patients’ mortality prediction and did not consider other important factors that might be encountered in day-to-day practice such as the rebleeding rate or surgical interventions.

The data we used were retrospectively collected from our registry which suggests heterogeneous information.

## Conclusion

Our study suggests that a machine learning program based on the available pre-endoscopic bleeding scores might provide a more accurate prediction for patients’ mortality rate after NVUGIB admission. By combining the results of the five scores in a ML algorithm, our tool might be considered useful, not only for endoscopists but also for emergency physicians to assess patients’ prognosis at their presentation. While our single-center study may not be sufficient to validate and implement this tool, it may be a starting point for future integration in the healthcare system.

## Data availability statement

The raw data supporting the conclusions of this article will be made available by the authors, without undue reservation.

## Ethics statement

The Ethics Committee of the University of Medicine and Pharmacy of Craiova, Romania approved this retrospective study and informed consent from all patients were acquired in the County Hospital before patient enrolment in the study (11977/24.03.2020). The patients/participants provided their written informed consent to participate in this study.

## Author contributions

BSU and AT-S: project design and manuscript writing. BSU, DNF, SI, SMC, and VFI: data collection. AT-S: data analysis. BSU, DIG, and IR: revise the manuscript and interpretation of data. All authors read and approved the final manuscript.
